# Possible involvement of sialidase and sialyltransferase activities in a stage-dependent recycling of sialic acid in some organs of type 1 and type 2 diabetic rats

**DOI:** 10.3389/fendo.2024.1289653

**Published:** 2024-06-24

**Authors:** Osas Graham Erhabor, Peter Obochi, Murtala Bindawa Isah, Mohammed Aliyu Usman, Ismaila Alhaji Umar, Mthokozisi B. C. Simelane, Mohammed Nasir Shuaibu, Md. Shahidul Islam, Mohammed Auwal Ibrahim

**Affiliations:** ^1^ Department of Biochemistry, Ahmadu Bello University, Zaria, Nigeria; ^2^ Department of Biochemistry, Umaru Musa Yar’adua University, Katsina, Nigeria; ^3^ Department of Biochemistry, University of Johannesburg, Johannesburg, South Africa; ^4^ School of Life Sciences, University of KwaZulu-Natal, Durban, South Africa

**Keywords:** sialic acid, sialidase, sialoconjugates, sialyltransferase, type 1 diabetes, type 2 diabetes

## Abstract

**Background:**

Type 1 (T1D) and type 2 (T2D) diabetes lead to an aberrant metabolism of sialoglycoconjugates and elevated free serum sialic acid (FSSA) level. The present study evaluated sialidase and sialyltranferase activities in serum and some organs relevant to diabetes at early and late stages of T1D and T2D.

**Methods:**

Sialic acid level with sialidase and sialyltransferase activities were monitored in the serum, liver, pancreas, skeletal muscle and kidney of diabetic animals at early and late stages of the diseases.

**Results:**

The FSSA and activity of sialidase in the serum were significantly increased at late stage of both T1D and T2D while sialic acid level in the liver was significantly decreased in the early and late stages of T1D and T2D, respectively. Furthermore, the activity of sialidase was significantly elevated in most of the diabetes-relevant organs while the activity of sialyltransferase remained largely unchanged. A multiple regression analysis revealed the contribution of the liver to the FSSA while pancreas and kidney contributed to the activity of sialidase in the serum.

**Conclusions:**

We concluded that the release of hepatic sialic acid in addition to pancreatic and renal sialidase might (in)directly contribute to the increased FSSA during both types of diabetes mellitus.

## Graphical Abstract

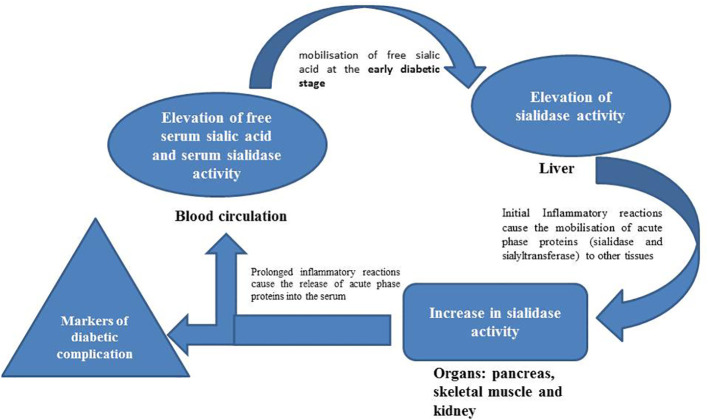

## Introduction

Diabetes mellitus is a metabolic disorder associated with increased blood glucose levels due to impaired pancreatic insulin secretion or action. The International Diabetes Federation (IDF) classifies the disease into two major types viz; Type 1 (T1D) and Type 2 Diabetes (T2D). The T1D is associated with absence of circulating insulin and the inability of pancreatic β-cells to express insulinogenic stimuli response resulting in hyperglycemia. Although T1D accounts for less than 10% of the 451 million diabetes patients (1, 2), it has continually been an important health menace because it relates to heredity. The T2D accounts for >90% of all the diabetic cases and it is associated with both insulin resistance and pancreatic β-cell dysfunction resulting to chronic hyperglycemia. Although the two types of diabetes result in hyperglycemia, the pathogenesis, pathophysiology and some complications associated with the two forms of the disease are quite distinct. Therefore, it is imperative to pursue independent scientific enquiries for the two types of diabetes.

Sialic acids are negatively charged nine carbon amino sugars that mainly exist at terminal end of glycoproteins and glycolipids where they mediate a number of vital functions including immunity, repulsiveness and cell to cell recognition (3). Elevated serum level of sialic acid has been repeatedly demonstrated as a key feature of diabetes mellitus in humans, and has been considered as a risk for the disease and related complications (4–6). Specifically, high levels of serum sialic acid were reported in T1D and T2D patients from India (6, 7), Pakistan (8), Trinidad and Tobago (9), Sweden (5) and England (10) for both patients with or without diabetic complications. Elevated plasma sialic acid was shown to be strongly associated with microvascular complications in T1D (4). In type 1 diabetic dogs, the elevated plasma sialic acid was also a potent biomarker (11). In rodents, the sialic acid-containing gangliosides of heart, skeletal muscle, liver, brain, kidney, spleen and pancreas decreased in alloxan induced diabetes (12). A different study reported a decrease in gangliosides of skeletal muscle of rats with STZ-induced T1D model, but an increase in animals with T2D two weeks after diabetes induction (13). Pari and Rothinam (14) reported a decrease in total sialic acid content of the liver and kidney of rats induced with T2D while Ibrahim et al. (15) reported an increase in total sialic acid content of the organs in rats with insulin resistance or hyperglycemia induced by high fructose or high glucose feeding respectively. Furthermore, elevated levels of sialic acid and/or sialylation were reported in the kidney (16), immunoglobulins (17) and erythrocytes (18) of patients with diabetes mellitus. It seemed therefore that the T1D/T2D - sialic acid nexus is a complex phenomenon that might be dependent on the type and stage of diabetes as well as the nature of the organism.

In mammals, the *de novo* biosynthesis of sialic acid is related to the hexosamine pathway whose metabolic flux is also increased during T2D. However, sialylation of glycoconjugates catalyzed by sialyltransferases is the major event that occurred after the biosynthesis. On the other hand, breakdown of glycoconjugates usually involves the hydrolysis of sialic acid by neuraminidases (sialidases). The hydrolyzed sialic acid is mostly returned to the cytosol and serves as a substrate for sialyltransferases for the biosynthesis of newer glycoconjugate molecules or further degradation (19). Indeed, mammalian sialidase (Neu1) was shown to play an important biological role in the development of insulin resistance through cellular signaling processes (20). Moreover, neuraminidase activity was also shown to increase in type 2 diabetic patients with or without cardiovascular complications (21) in addition to other organs of diabetic rats (12, 22). These enzymes may thus be linked to some of the pathophysiological features of diabetes mellitus. In our recent study, we also observed an increase in the mRNA expression of *NEU1* gene in some organs of T2D rats while the gene expression of UDP-N-acetylglucosamine-2-epimerase/N-acetylmannosamine kinase (*GNE*) was elevated in the pancreas, but not other organs (23). These suggest that the increased sialic acid level in the organs (except for pancreas) of diabetic rats could not be due to increased endogenous synthesis of sialic acid but probably due to increased recycling of glycoconjugates. Collectively, the sialyltransferases and sialidases enable the recycling of sialic acid within the mammalian systems (19) and this appears to be the main modulatory point for the observed sialic acid changes across cells and tissues during diabetes mellitus.

Herein, we monitored sialidase and sialyltransferase activities in the serum and some diabetes-relevant organs (liver, pancreas, skeletal muscle, and kidney) at the early and late stages of both T1D and T2D rat model, as well as attempted to decipher the relationship between the enzymes and total sialic acid across the organs during the disease. This might provide the possible role of hydrolysis (sialidase) and/or transfer (sialyltranferase) of sialic acid as key mediators to the observed sialic acid metabolism dysregulation within many organs of diabetic animals. Understanding the modulation of these enzymes in relation to sialic acid changes in T1D and T2D could also afford some unique molecular insights into the biological processes responsible for the increased sialic acid in the organs of diabetic animals. Additionally, the information would further deepen our current understanding of the pathophysiology of diabetes mellitus, especially with respect to glycobiology.

## Materials and methods

### Chemicals and reagents

Thiobarbituric acid, Streptozotocin (STZ), asialofetuin, cytidine 5’ monophosphate-N-acetylneuraminic acid (CMP-sialic acid), fetuin, standard sialic acid and DEAE-Cellulose were procured from Sigma Chemical Company, USA while rat insulin ELISA kit was purchased from Wkea Med Supplies Corporation, China. Sodium arsenite from Hopkin and Williams Ltd., England while sodium periodate was procured from BDH Chemicals, Poole, England.

### Experimental animals and induction of T1D and T2D

A total of 58 apparently healthy male Wistar rats with a body weight range of 150–200 g were obtained from the Department of Pharmacology and Therapeutics, Ahmadu Bello University, Zaria, Nigeria, and kept in well-ventilated laboratory cages in the animal house at room temperature. Animals were supplied with commercial rat chow (Vital Feeds, Jos, Nigeria) and drinking water *ad libitum*. Prior to the experiment, the animals were also allowed to acclimatize for a week and maintained according to the guidelines of Ahmadu Bello University Committee on Animal Use and Care (ABUCAUC) with an approval number of ABUCAUC/2018/006.

The animals were grouped into three (3); Normal Control group, NC, (21 animals) and Type 1 Diabetic Group (16 animals) and Type 2 Diabetic Group (21 animals). The T1D group animals were induced with diabetes by injecting a single intraperitoneal dose (60 mg/kg body weight) of STZ dissolved in citrate buffer (100 mM, pH 4.5) to overnight fasted animals (24) while the T2D group were induced with T2D by a prior feeding of fructose solution *ad libitum* (10%) for 2 weeks for induction of insulin resistance which was followed by a similar injection of a low dose (40 mg/kg body weight) of the STZ for the induction of partial pancreatic β-cell dysfunction (25). The rats in the NCG were given drinking water and intraperitoneally administered with 100 mM citrate buffer instead of 10% fructose and STZ injection, respectively.

The T1D animals with non-fasting blood glucose (NFBG) >400 mg/dL and T2D animals with NFBG within 200 – 400 mg/dL were considered diabetic (26), 7 days after the STZ injection. For further confirmation of T2D, five animals were removed from the NCG and T2D group each, fasted overnight and the blood collected by cardiac puncture to investigate the FBG level by using a portable glucometer (Glucoplus Inc., Saint-Laurent, Quebec, Canada) and fasting serum insulin level by an enzyme-linked immunosorbent assay (ELISA) method using a rat insulin ELISA kit (Wkea Med Supplies Corporation, China) as described by the manufacturer. This was done to allow the computation of homeostasis model assessment (HOMA-IR and HOMA-β) scores in order to ascertain the degree of insulin resistance and β- cells damage for the two groups one week after the diabetes induction in the respective groups. The calculation of the HOMA scores was done using the following formulas:


$$\text{HOMA}-\text{IR}=\frac{\text{Fasting serum insulin in}\frac{\text{UL}}{\;}\text{X Fasting blood glucose in mmol}/\text{L }}{22.5}$$

$$\text{HOMA}-\text{β}=\frac{20\text{ X Fasting serum insulin U}/\text{L}}{\text{Fasting blood glucose inmmol}/\text{L}-3.5\;}$$

Conversion factor: insulin (1U/L = 7.174 pmol/L).

The experiment lasted for 9 weeks and during the period, weekly NFBG levels of rats in all the three experimental groups were monitored from the blood collected via the tail vein.

### Collection and preparation of blood and organs homogenates

For the early stage of T1D and T2D, eight (8) animals from each of the three experimental groups were euthanized three weeks after the onset of T1D and T2D, and the whole blood from each experimental animal was collected by cardiac puncture. Thereafter, these blood samples were centrifuged (920 × g, 15 min) to obtain the serum from each blood sample which were used for sialic acid, sialidase and sialyltransferase assays. The liver, pancreas, skeletal muscle and kidney samples were also removed aseptically from each animal, and immediately kept in -20°C freezer until needed. The same procedure was repeated at week 9 after the onset of T1D and T2D using the remaining 8 animals in each group and was considered as the late stage of the disease. The selection of the 3^rd^ and 9^th^ weeks was based on the temporal dynamics of diabetes progression. The 3^rd^ week after diabetes induction confirmation was considered to be an early stage of the disease’s development, in line with previous studies on animal models (25). Similarly, the 9^th^ week represented a later stage in the progression of diabetes where additional changes in enzyme activities and glycosylation patterns might have occurred.

For the sialic acid and sialidase assays, each organ (0.5 g) was homogenized in 2.5 mL of phosphate buffer saline (50 mM) and the resulting mixture was divided into 2 equal parts. One of these homogenate preparations was centrifuged (2058 × g, 50 min), and the supernatant was immediately used for sialidase activity assay. However, the other homogenate preparation was mixed with equal volume of 0.1 M H_2_SO_4_ and incubated at 80 °C in a water bath for one hour for optimal hydrolysis of the bonded sialic acid. At the end of the incubation, the resulting hydrolysates were also centrifuged (102 × g, 15 min) and the supernatant was collected and used for sialic acid assay. For sialyltransferase assay, each organ (0.5 g) was also homogenized in 1.5 mL Tris-HCl buffer (0.1 M, pH 6.8) containing 2-mercaptoethanol (2 mM) and 0.1% Triton X-100. The homogenates were also centrifuged (9125 × g, 1 h) and the resultant supernatants were removed and filtered through a glass wool to obtain filtrates which were then used for the analysis of sialyltransferase activity.

### Sialic acid analysis

The sialic acid level of each of the organ and serum samples was analyzed by the thiobarbituric acid assay (TBA) method. Exactly 500 µL aliquots of sample containing sialic acid (tissue homogenates and serum) was added to periodate solution (250 µL, 25 mM) and incubated at 37 °C for 30 min. Excess periodate was reduced with 2% arsenite solution (200 µL) and 70% thiobarbituric acid (2 mL) was added. The mixture was then incubated at 90 °C for 7.5 min. After cooling, acid butanol reagent (2.5 mL) was added and centrifuged (920 × g, 5 min) to allow the partitioning of two phases. Afterwards, the absorbance of the butanol layer was taken at 549 nm from where the sialic acid concentration in each of the samples was calculated from a standard curve of sialic acid.

### Sialidase assay

The activity of sialidase was analyzed by monitoring the release of sialic acid from fetuin as described by Aminu et al. (27). Here, the serum or homogenates (100 μL) was incubated with fetuin (50 μL, 1 mg/mL) in acetate buffer (pH 5.9) at 37°C for 30 min. The sialic acid released from the fetuin was then quantified using TBA assay method as earlier described. To account and zero the effects of non-specific degradation of the substrate, a control tube was set up alongside the experimental samples. This control tube contained all the experimental reagents except the samples. One unit of sialidase activity was defined as the amount of enzyme that hydrolyzed 1 μmol of sialic acid from fetuin per min under standard assay conditions.

### Sialyltransferase assay

The activity of sialyltransferase was determined by evaluating the transfer of sialic acid from the CMP-sialic acid to asialo-fetuin (28). Each assay mixture contained 25 μL of each of the followings; Tris-HCl buffer (0.2M, pH 6.5) containing 0.1 M MnCl_2_ and 0.1% Triton X100, bovine serum albumin (5 mg/mL), asialofetuin (1 mg/mL) dissolved in 0.85% NaCl, CMP-sialic acid (100 μg/mL) and the serum or organ homogenate. In the control set up, the acceptor substrate (asialofetuin) was replaced with 0.2 M Tris-HCI (pH 6.5). The reaction mixture was incubated for 30 min at 37°C and diluted with 1 mL of 5mM sodium phosphate buffer (pH 6.9). The mixture was then immediately made to pass through a pre-equilibrated DEAE-cellulose column (4.95 mm x 10.7 cm) and eluted with 750 μL of 5 mM phosphate buffer (pH 6.9). The traces of free sialic acid and unreacted CMP-sialic acid were trapped by the column. To each eluate, 170 μL of 1 M H_2_SO_4_ was added and the mixture was heated for 1 h at 80°C to hydrolyze the sialyl-conjugates (sialofetuin) and the sialic acid released from the conjugates was then determined using TBA assay method as earlier described. One unit of sialyltransferase activity was defined as the amount of enzyme that incorporated 1 μmol of sialic acid from CMP-sialic acid to asialofetuin per minute under standard assay conditions.

### Statistical analysis

Data was analyzed using Statistical Package for Social Sciences (SPSS) version 20 (IBM Corporation, NY, USA). Multiple regression analysis was performed and Pearson correlation test was used for correlation analysis. Statistical difference between enzymes in serum and organs were analyzed using one way analysis of variance (ANOVA). P values less than 0.05 were considered as significant.

## Results

**Figure 1:**
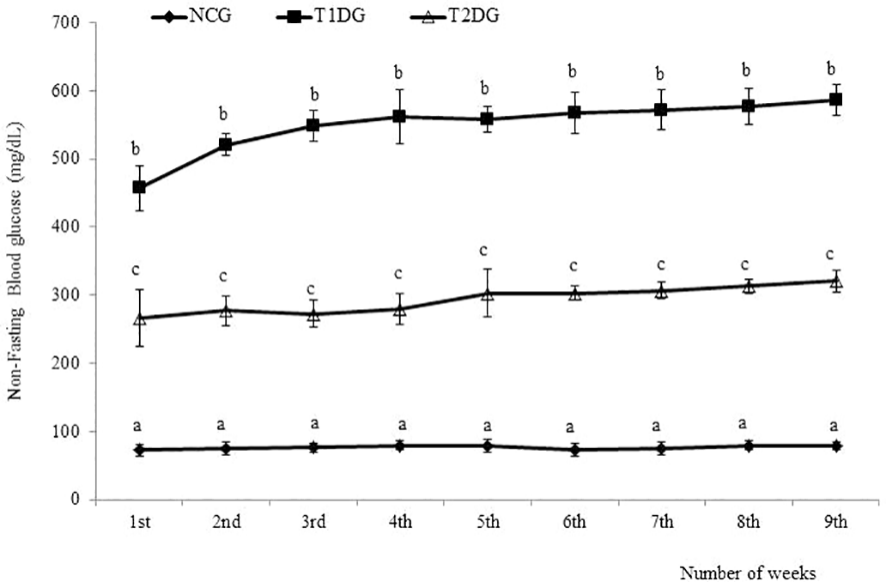
The weekly non-fasting blood glucose levels of type 1 and type 2 diabetic rats in relation to normal control. Data for each group are presented as mean ± standard deviation of 16 and 8 animals for 1 - 3 and 4-9 weeks intervals, respectively. NCG: Normal Control Group, T1DG: Type 1 Diabetic Group, T2DG: Type 2 Diabetic Group. Different alphabets within a week indicate significant difference (*P* < 0.05).

The two most prominent characteristics of T2D pathogenesis (insulin resistance and partial pancreatic β cells dysfunction) were successfully induced in the rats of T2D group as demonstrated by the significantly higher HOMA-IR scores (*P*< 0.05) in addition to the significantly lower HOMA-β scores (*P*< 0.05) among the T2D group compared to the NCG (**Table 1**). The NFBG levels monitored over the 9-week experimental period revealed a progressive and significant increase (*P*<0.05) in both T1D and T2D groups compared to NCG. Within the diabetic groups, T1D group had a significantly (*P*<0.05) higher NFBG which was maintained at > 400 mg/dL while the NFBG of T2D group was maintained between 200–300 mg/dL throughout the 9-week experimental period (**Figure 1**).

**Table 1:** Homeostasis model assessment scores of insulin resistance (HOMA-IR) and β-cell function (HOMA- β) for the normal and type 2 diabetic animals one week after diabetes induction.

Homeostasis Model Assessment	NCG	T2DG
HOMA-IR	1.82±0.25^a^	8.74±2.19^b^
HOMA-β	555.12±131.39^a^	15.22±3.59^b^

Data are presented as the mean ± SD of five animals. ^a-b^Different superscript letters along a row indicate significant difference at p <0.05. NCG: Normal Control Group; T2DG: Type-2 Diabetic Group.

HOMA-IR = [(Fasting serum insulin in U/L x Fasting blood glucose in mmol/L) / 22.5].

HOMA-β = (Fasting serum insulin in U/L x 20 / Fasting blood glucose in mmol/L – 3.5).

Conversion factor: insulin (1U/L= 7.174 pmol/L).

At the early stage (3 weeks) of the disease, the diabetic groups had a statistically similar (*P*> 0.05) free serum sialic acid (FSSA) with NCG but a significantly higher FSSA (*P*< 0.05) was observed in the two diabetic groups at the late stage (9-week) of the disease. The FSSA was significantly higher (*P*< 0.05) in T1D group compared to the T2D group (**Table 2**). Similarly, serum sialidase activity was not affected at the early stage of both types of diabetes but a significant increase was observed at the late stage of diabetes compared to NCG (*P*< 0.05). Although the T1D group had a higher serum sialidase activity than the T2D group, the difference was statistically insignificant (*P*>0.05). In contrast to the FSSA level and sialidase activity, the serum sialyltransferase activity was neither affected by the type or stage of the diabetes mellitus (**Table 2**).

**Table 2:** The effects of type 1 and type 2 diabetes on the free serum sialic acid levels and sialidase and sialyltransferase activities in rats.

	Sialic acid (mg/mL)		Sialidase (µmol/min)		Sialyltransferase (µmol/min)	
	3^rd^ week	9^th^ week	3^rd^ week	9^th^ week	3^rd^ week	9^th^ week
NCG	0.54 ±0.19	0.56±0.10^a^	32.68±2.76	28.32±1.84^a^	46.12 ±2.10	46.85±3.01
T1DG	0.56±0.01	0.86±0.08^b^	27.03± 4.33	48.58± 3.31^b^	47.59± 3.93	48.83± 3.95
T2DG	0.63±0.01	0.75±0.09^b^	32.85±4.33	44.98±1.72^b^	50.46±2.39	50.00±5.08

Data are presented as the mean ± SD of eight animals. ^a-b^Different superscript letters along a row indicate significant difference at P< 0.05. NCG: Normal Control Group; T2DG: Type-2 Diabetic Group.

**Figure 2:**
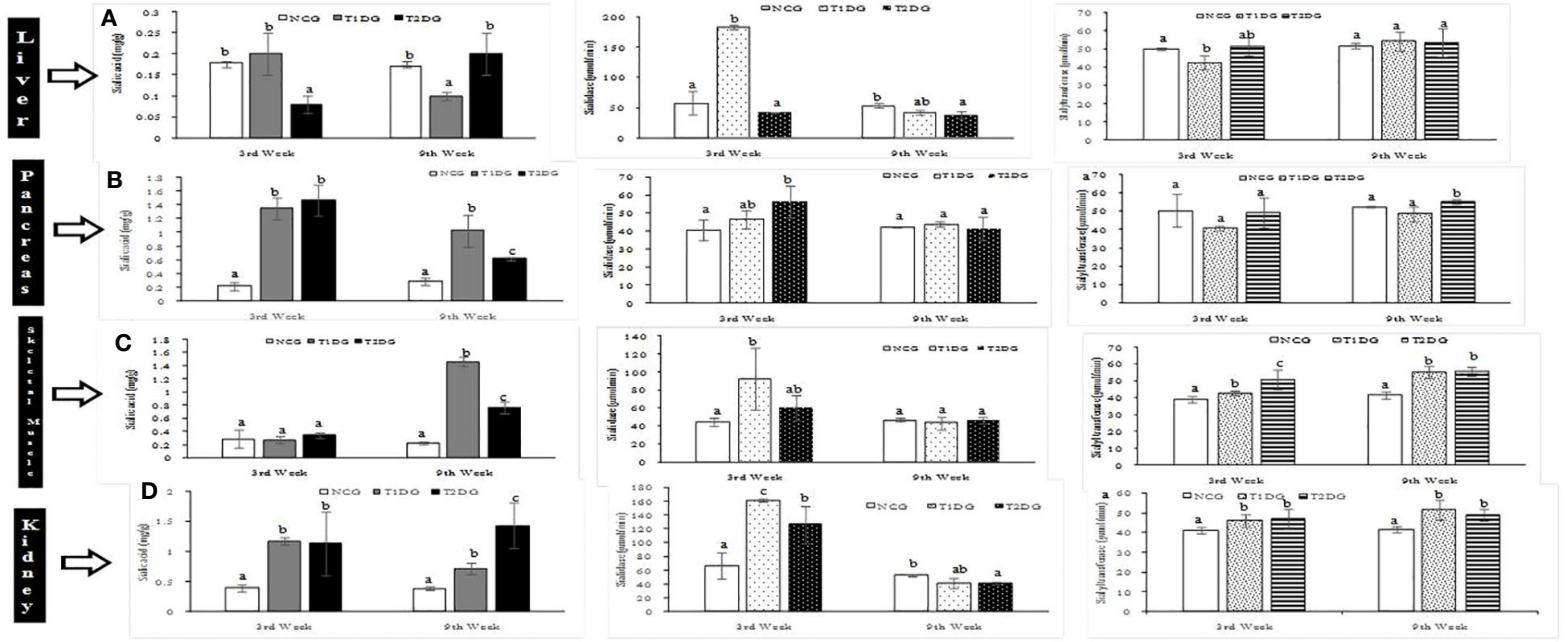
Sialic acid distribution with sialidase and sialyltransferase activities of liver **(A)**, pancreas **(B)**, skeletal muscle **(C)** and kidney **(D)** of type 1 and type 2 diabetic rats at early (3^rd^ week) and late (9^th^ week) stages. Data are presented as mean ± standard deviation of 8 animals. NCG: Normal Control Group, T1DG: Type 1 Diabetic Group, T2DG: Type 2 Diabetic Group. Different alphabets within a week indicate significant difference (*P* < 0.05).

In the liver, the T1D did not affect the total sialic acid level at the first 3 weeks, but led to a significant reduction (*P*>0.05) in the total sialic acid level at week 9. Conversely, the T2D induced a significant decrease in the liver total sialic acid level which was reversed at the 9^th^ week (**Figure 2A**). With respect to sialidase activity, an opposing pattern was observed at the early stage, where the enzyme activity was significantly elevated in the T1D group (*P*<0.05) whilst a significant reduction was recorded in the T2D group (*P*<0.05). However, both T1D and T2D caused a significant reduction in sialidase activity at the late stages. Meanwhile, the activity of sialyltransferase in the liver was significantly decreased (*P*<0.05) only at the early stage of T1D which was reversed at the late stage while T2D did not affect sialyltransferase activity at both early and late stages. On the other hand, both T1D and T2D significantly increased the pancreatic total sialic acid at the early stages (*P*<0.05) and the pattern was maintained at the late stage of the experiment (**Figure 2B**). Pancreatic sialidase activity was significantly increased (*P*<0.05) at the early stage of T2D while sialyltransferase activity was increased at the late stage only. The T1D had no effect on pancreatic sialidase activity at both stages but significantly decreased (*P*<0.05) sialyltransferase activity at the early stage (**Figure 2B**).

The total sialic acid level of skeletal muscle was not affected by both T1D and T2D at the early stage of the diseases but the levels were significantly increased (*P*<0.05) at the late stage with the T1D causing a more profound increase in the total sialic acid level in the skeletal muscle (**Figure 2C**). In contrast, both T1D and T2D significantly increased (*P*<0.05) the sialidase and sialyltransferase activities at the early stage but in the case of T1D, there was reversal of sialidase activity to near-normal, at the late stage (**Figure 2C**). In the kidney, the total sialic acid content and sialidase activity were significantly increased (*P*<0.05) by both T1D and T2D at the early stage and the pattern was maintained at the late stage in the case of sialic acid level but a reversal of sialidase activity to normal level occurred at the late stage (**Figure 2D**). The renal sialyltransferase activity was also significantly (*P*>0.05) increased in the two types of diabetes mellitus at both early and late stages of T1D and T2D (**Figure 2D**).

Multiple regression analysis was conducted to identify the possible contribution of the various organs to the serum total sialic acid level and activities of sialidase and sialyltransferase. Serum total sialic acid level was strongly and significantly associated with liver total sialic acid level of T2D group at the late stage (R^2^ = 0.971; *P*= 0.015) whereas the regression was strong but not statistically significant in T1D group (R^2^ = 0.856; *P* = 0.075) (**Table 3**). On the other hand, the possible contribution of pancreas to serum sialidase activity in T1D group at the late stage was identified (R^2^ = 0.907; P = 0.048), whereas in T2D group, the possible contribution of the kidney at the early stage was identified (R^2^ = 0.842; *P* = 0.004). There was a moderate regression between serum sialidase activity and sialidase activity in the kidney of T1D group (R^2^ = 0.595; *P* = 0.228). Serum sialyltransferase activity was dependent on liver sialyltransferase activity at the late stage in both T1D (R^2^ = 0.937; *P* = 0.032) and T2D groups (R^2^ = 0.980; *P* = 0.001). In addition, sialyltransferase activity in the skeletal muscle of T1D group was strongly and significantly associated with serum sialyltransferase activity at the late stage (R^2^ = 0.957; *P* = 0.013). Correlation analysis of FSSA level with the sialidase and sialyltransferase activities in the serum revealed a non-significant (*P*>0.05) negative correlation at early stages for the two types of diabetes, except sialyltransferase activity at the early stage of T2D which was significant (r=-0.846; *P*=0.034) (**Table 4**). At later stages of both types of diabetes, the correlation was positive and statistically non-significant (*P*>0.05).

**Table 3:** Multiple regression analysis of sialic acid level and sialidase/sialylytransferaseactivities of various organs associated with the corresponding serum parameters.

	Type of diabetes	Organ	Week after induction	R^2^	P-value
Serum sialic acid vs organ sialic acid	T1D	Liver	3^rd^	0.856	0.075
	T2D	Liver	9^th^	**0.971**	**0.015**
Serum sialidase activity vs organ sialidase activity	T1D	Pancreas	9^th^	**0.907**	**0.048**
	T1D	Kidney	9^th^	0.595	0.228
	T2D	Kidney	3^rd^	**0.842**	**0.004**
	T2D	Skeletal muscle	9^th^	0.616	0.116
Serum sialyltransferase activity vs organ sialyltransferase activity	T1D	Liver	9^th^	**0.937**	**0.032**
	T1D	Skeletal muscle	3^rd^	0.664	0.185
	T1D	Skeletal muscle	9^th^	**0.957**	**0.013**
	T2D	Liver	9^th^	**0.980**	**0.001**

Multiple regression analysis was carried out using Graphpad Prism and only analyses with R^2^> 0.5 (moderate to strong associations) were shown in this table. Total sialic acid levels, sialidase and sialyltransferase activities of individual organs (liver, pancreas, skeletal muscle and kidney) were used as predictors (independent variables) of the serum total sialic acid level, and sialidase/sialyltransferase activities. Values in bold are statistically significant (P < 0.05).

**Table 4:** Correlation analysis serumsialic acid level and serum sialidase/sialyltransferase activities.

	Sialidase				Sialyltransferase			
	3rd week		9th week		3rd week		9th week	
	r	p-value	r	p-value	r	p-value	r	p-value
T1DG	-0.603	0.397	0.792	0.208	-0.709	0.291	-0.011	0.989
T2DG	0.072	0.892	0.799	0.411	-0.846	0.034*	0.554	0.626

## Discussion

Both T1D and T2D are associated with increased output of sialic acid in the serum and some organs in humans and experimental animals (5–7, 11). Increased *de novo* biosynthesis measured as the mRNA expression level of the rate determining enzyme (*GNE*), might not account for the observed increase of sialic acid level in most organs of diabetic animals (23). In this study, we reported that the activities of sialidase and sialyltransferase are modulated in serum and selected organs of diabetic rats and could be involved in the recycling of sialic acid depending on the organ, stage and type of diabetes.

Although previous reports have demonstrated an increase in FSSA during diabetes, it was evident from this study that the increase could be stage specific because there was no increase at the early phases of both types of diabetes. Moreover, sialidase was also unaffected at the early phase of the diseases but the activity was enhanced at the late stage, which corroborates with the observed pattern for FSSA. The actual source of elevated FSSA in diabetes has not been established with certainty, but endothelial damage has been implicated. Indeed, vascular endothelium is associated with high level of sialic acid; therefore, tissue injury during the diabetes may lead to the observed activation of sialidase (29), especially the GPI-anchored acidic form of sialidase which was increased in the erythrocyte membrane of diabetic patients (18). It has been established that diabetes leads to increase in serum sialic acid-containing gangliosides (30–32). In response to the increase, cell surface associated sialidase (NEU3) may be secreted from the cell surface as hypothesized in cancer (31, 33), which could account for the elevated serum sialidase activity in our study. Furthermore, from the findings of the present study, the pancreas may contribute to circulating serum sialidase at late stages of T1D, probably as a result of leakage due to prolonged and more severe oxidative insults and pancreatic β-cell death in T1D. This could be supported by the higher activity of the enzyme in the serum of T1D group compared to T2D group. In addition to the contribution of circulating sialidase in the serum, sialic acid release from the liver may contribute to the elevated FSSA in the diabetic rats as suggested by the regression analysis (**Table 3**).

The concomitant increase in total sialic acid content of the organs as well as sialidase and sialyltransferase activities may indicate an active recycling of exogenous sialoconjugates. It is noteworthy that the activity of sialyltransferase decreased or did not change only in the pancreas of the diabetic rats (**Figure 2**), despite the significant increase in pancreatic total sialic acid content. This indicates that elevated pancreatic sialic acid level may not be due to the influx of exogenous sialoglycoconjugates. In our previous study (23), the GNE gene expression was elevated in the pancreas of diabetic animals with a concomitant elevation in total sialic acid content of the organ. Since it has been previously demonstrated that sialyltransferases are insensitive to the rate of flux through the sialic acid biosynthetic pathway (34), the data in the present study also support increased endogenous synthesis of sialic acid by the pancreas rather than recycling of exogenous sialic acid.

Cohen-Forterre et al. (22) showed fluctuations in bound sialic acid content, sialidase and sialyltransferase activities in the kidney cortex of spontaneously-diabetic and STZ-induced diabetic rats. In the spontaneously diabetic rats with three to five weeks diabetes, sialidase activity was elevated while sialyltransferase activity was unchanged in the kidney cortex compared to age-matched controls. There was a reduction in the amount of bound-sialic acid in the report by Cohen-Forterre et al. (22) while in our analysis, the total sialic acid content of the kidney increased early. Herein, there was also a significant elevation in sialidase and sialyltranferase activities in the kidney of the rats. The increase in the total sialic acid content of the kidney despite the increase in sialidase and sialyltransferase activities at the early stage of diabetes (when serum sialic acid content has not yet significantly changed) may indicate that the kidney modulates its sialylation pattern at the early stage of diabetes.

In conclusion, there was an increase in FSSA level that manifests at the late stages of experimental T1D and T2D. The increase in the FSSA level may be partially contributed by increased sialic acid content of the liver and increased circulating sialidase. Damage to the pancreas and kidney caused by diabetes may cause further elevation in serum sialidase activity. The liver and skeletal muscle may be responsible for activity of sialyltransferase activity in the serum. Whether these observations are true in diabetic patients need to be carefully investigated due to the complex interplay between various organs and etiologic factors in the metabolism of sialic acid and sialoglycoconjugates. Additionally, our future study would focus on the enzymatic roles and gene expression changes of individual isozymes of both sialidases and sialyltransferase during TID and T2D.

## Data availability statement

The original contributions presented in the study are included in the article/supplementary material. Further inquiries can be directed to the corresponding author.

## Ethics statement

The animal study was approved by Ahmadu Bello University Committee on Animal Use and Care (ABUCAUC). The study was conducted in accordance with the local legislation and institutional requirements.

## Author contributions

OE: Formal analysis, Investigation, Methodology, Writing – original draft. PO: Formal analysis, Investigation, Methodology, Writing – original draft. MI: Conceptualization, Formal analysis, Investigation, Writing – original draft. MU: Methodology, Writing – review & editing. IU: Methodology, Resources, Supervision, Writing – review & editing. MS: Formal analysis, Investigation, Resources, Writing – review & editing. MNS: Formal analysis, Methodology, Resources, Supervision, Writing – review & editing. MSI: Formal analysis, Funding acquisition, Investigation, Supervision, Writing – review & editing. MAI: Conceptualization, Data curation, Formal analysis, Funding acquisition, Investigation, Methodology, Project administration, Resources, Supervision, Writing – original draft.
